# Elucidating afferent-driven presynaptic inhibition of primary afferent input to spinal laminae I and X

**DOI:** 10.3389/fncel.2022.1029799

**Published:** 2023-01-11

**Authors:** Volodymyr Krotov, Kirill Agashkov, Sergii Romanenko, Oleh Halaidych, Yaroslav Andrianov, Boris V. Safronov, Pavel Belan, Nana Voitenko

**Affiliations:** ^1^Department of Sensory Signaling, Bogomoletz Institute of Physiology, Kyiv, Ukraine; ^2^Department of Molecular Biophysics, Bogomoletz Institute of Physiology, Kyiv, Ukraine; ^3^i3S-Instituto de Investigação e Inovação em Saúde, Universidade do Porto, Porto, Portugal; ^4^Neuronal Networks Group, Instituto de Biologia Molecular e Celular, Universidade do Porto, Porto, Portugal; ^5^Department of Biomedicine and Neuroscience, Kyiv Academic University, Kyiv, Ukraine; ^6^Dobrobut Academy Medical School, Kyiv, Ukraine

**Keywords:** presynaptic inhibition, spinal cord, lamina I, lamina X, primary afferents, A-fibers, C-fibers

## Abstract

Although spinal processing of sensory information greatly relies on afferent-driven (AD) presynaptic inhibition (PI), our knowledge about how it shapes peripheral input to different types of nociceptive neurons remains insufficient. Here we examined the AD-PI of primary afferent input to spinal neurons in the marginal layer, lamina I, and the layer surrounding the central canal, lamina X; two nociceptive-processing regions with similar patterns of direct supply by Aδ- and C-afferents. Unmyelinated C-fibers were selectively activated by electrical stimuli of negative polarity that induced an anodal block of myelinated Aβ/δ-fibers. Combining this approach with the patch-clamp recording in an *ex vivo* spinal cord preparation, we found that attenuation of the AD-PI by the anodal block of Aβ/δ-fibers resulted in the appearance of new mono- and polysynaptic C-fiber-mediated excitatory postsynaptic current (EPSC) components. Such homosegmental Aβ/δ-AD-PI affected neurons in the segment of the dorsal root entrance as well as in the adjacent rostral segment. In their turn, C-fibers from the L5 dorsal root induced heterosegmental AD-PI of the inputs from the L4 Aδ- and C-afferents to the neurons in the L4 segment. The heterosegmental C-AD-PI was reciprocal since the L4 C-afferents inhibited the L5 Aδ- and C-fiber inputs, as well as some direct L5 Aβ-fiber inputs. Moreover, the C-AD-PI was found to control the spike discharge in spinal neurons. Given that the homosegmental Aβ/δ-AD-PI and heterosegmental C-AD-PI affected a substantial percentage of lamina I and X neurons, we suggest that these basic mechanisms are important for shaping primary afferent input to the neurons in the spinal nociceptive-processing network.

## 1. Introduction

Presynaptic inhibition (PI) of primary afferents is a fundamental mechanism shaping their input to the spinal cord. In the proprioceptive network, feedback inhibition ensures smoothness of locomotion (Fink et al., 2014), while PI of nociceptive afferents is critically important for processing pain signals (Melzack and Wall, 1965; Fernandes et al., 2020; Comitato and Bardoni, 2021; Ramírez-Morales et al., 2021; Krotov et al., 2022). PI can be induced by supraspinal descending fibers or segmental primary afferents and is mediated by GABA release at central terminals of primary afferents causing Cl^–^ efflux and primary afferent depolarization (PAD) (Guo and Hu, 2014; Comitato and Bardoni, 2021). PAD either prevents invasion of action potentials to the afferent terminal or reduces their amplitude, thus diminishing glutamate release to spinal neurons (Rudomin and Schmidt, 1999; Willis, 1999).

Despite its obvious physiological relevance, AD-PI and its effect on the afferent input to the spinal nociceptive neurons remain poorly understood. PI was mostly examined indirectly by morphological analysis of the axo-axonic synapses at primary afferent terminals, by recording the antidromic passive spread of PAD to the dorsal root [dorsal root potential (DRP)], or by analyzing changes in excitability of the intraspinal afferents by conditioning stimulation of cutaneous nerves (Hentall and Fields, 1979; Fitzgerald and Woolf, 1981; Calvillo et al., 1982). Recently developed methods that involve genetic manipulations to knock down GABA_A_ receptor subunits at primary afferent terminals (Chen et al., 2014) or to express light-sensitive ChR2 receptors for activation of specific populations of GABAergic neurons (Fink et al., 2014; François et al., 2017) are time-consuming, sophisticated and expensive. Therefore, the development of simple approaches for investigating PI on a broad scale might greatly benefit spinal cord research. In the case of AD-PI, such method has recently been described (Fernandes et al., 2020). It relies on selective activation of C-fibers in the dorsal root through a suction electrode using electrical stimulus of inverted polarity. By applying this approach to the intact spinal cord preparations with several preserved dorsal roots, it was possible to reveal a homosegmental Aβ/δ-AD-PI and heterosegmental C-AD-PI of the C-fiber input to spinal lamina I neurons in the rat (Fernandes et al., 2020). In the present work, we used potential of this approach to further study the mechanisms of spinal processing of peripheral input.

The aim of this study was to examine AD-PI of peripheral inputs in rodent models, including the mouse, a species that nowadays is frequently used as a research model due to the abundance of its genetically modified strains. We did recordings from the neurons in the marginal zone, lamina I, and the area surrounding the central canal, lamina X, to examine effects for these two regions which are involved in spinal nociception and receive similar patterns of direct supply from cutaneous and visceral Aδ- and C-afferents (Light and Perl, 1979; Honda, 1985; Honda and Perl, 1985; Luz et al., 2015; Fernandes et al., 2016; Krotov et al., 2019). We have demonstrated that C-AD-PI is reciprocal for afferents from the L4 and L5 dorsal roots, controls the Aβ/δ- and C-fiber input and regulates spike discharge. We have also shown that primary afferent input to the nociceptive-processing spinal cord regions laminae I and X is similarly affected by AD-PI.

## 2. Materials and methods

### 2.1. Animals

In the present study, we used adult mice (2–3-month-old) and young rats (P11–13) of either sex. All experimental procedures were approved by the Animal Ethics Committee of the Bogomoletz Institute of Physiology (Kyiv, Ukraine) and performed in accordance with the European Commission Directive (86/609/EEC), ethical guidelines of the International Association for the Study of Pain, and the Society for Neuroscience Policies on the Use of Animals and Humans in Neuroscience Research.

### 2.2. *Ex vivo* spinal cord preparations

Lamina I neurons were studied in the isolated lumbosacral spinal cord preparations from adult mice using the approach described previously (Tadokoro et al., 2022). Briefly, a mouse was quickly decapitated, the vertebral column was cut out and immersed in oxygenated sucrose solution (20–22°C) containing (in mM): 200 sucrose, 2 KCl, 1.2 NaH_2_PO_4_, 0.5 CaCl_2_, 7 MgCl_2_, 26 NaHCO_3_, 11 glucose (pH 7.4 when bubbled with 95% O_2_ and 5% CO_2_). The lumbosacral cord with preserved unilateral L5 or L5 and L4 dorsal roots was dissected, cleaned from the dura/pia mater, and glued to the metal plate to position the lateral lamina I on top.

Lamina X neurons were studied using young rats (Krotov et al., 2017, 2022). After removing from the vertebral column and peeling the dura mater, the spinal cord was hemisected along the midline, and the half containing roots was glued (medial side up) to a metal plate for the recordings.

### 2.3. Recordings

The experiments were performed at room temperature (20–22°C) in oxygenated solution containing (in mM): NaCl 125, KCl 2.5, CaCl_2_ 2, MgCl_2_ 1, NaH_2_PO_4_ 1.25, NaHCO_3_ 26 and glucose 10 (pH 7.4, 95% O_2_ and 5% CO_2_).

Dorsal root potentials and compound action potentials (CAPs) were recorded with a suction electrode from the L4 or L5 dorsal root close to its entrance to the spinal cord. The electrodes filled with the bath solution had a resistance of 20–100 kΩ.

Lamina I and X neurons were visualized for the whole cell patch-clamp recordings using the oblique infrared LED illumination technique (Safronov et al., 2007; Szûcs et al., 2009). Patch pipettes pulled from borosilicate glass using a P-87 horizontal puller (Sutter Instruments, USA) had a resistance of 3–5 MΩ after filling with the solution of the following composition: 145 K-gluconate, 2.5 MgCl_2_, 10 HEPES, 2 Na_2_-ATP, 0.5 Na-GTP, and 0.5 EGTA (pH 7.3). Neurons were voltage clamped at −70 or −60 mV. Offset potentials were compensated before seal formation. Liquid junction potentials were not compensated.

MultiClamp 700B amplifier and Digidata 1320A/Digidata 1440 digitizers under the control of the pClamp software (Molecular Devices, CA, USA) were used for data acquisition. Signals were Bessel filtered at 2.6 kHz and sampled at 20 kHz. All chemicals were from Sigma-Aldrich (MO, USA).

### 2.4. Electrical stimulations

Dorsal roots (L4 and/or L5) were stimulated *via* suction electrodes connected to ISO-Flex (AMPI, Israel) stimulators, as described (Krotov et al., 2019; Fernandes et al., 2020). Positive pulses of current (+150 μA × 1 ms) were applied to activate all primary afferents, including high-threshold-Aδ- and C-afferents. Selective activation of C-fibers was achieved by applying pulses of negative polarity (−150 μA × 1 ms) which induced an anodal block of fast conducting Aβ/δ-fibers (Fernandes et al., 2020). Stimuli were applied at 0.1 Hz to avoid the slowing-down of conduction in C-fibers (Pinto et al., 2008b) and the wind-up phenomenon (Hachisuka et al., 2018).

The monosynaptic input was identified on the basis of the low failure rates (< 30%) and small latency variations (less than 2 ms) as described previously (Pinto et al., 2008b; Krotov et al., 2019). Afferent fibers mediating direct input were classified according to their conduction velocity (CV), which was calculated as the length of the root, from the opening of the suction electrode to the dorsal root entry zone, divided by the latency of the monosynaptic response with a 1 ms allowance for synaptic transmission. Fibers with CV below 0.5 m/s were considered as C-fibers (Pinto et al., 2008a). Afferents with CV ranging from 0.6 to 1.4 m/s were classified as Aδ-afferents. Monosynaptic inputs from faster-conducting Aβ-afferents [CV > 3.5 m/s, (Pinto et al., 2008a)] were observed only in lamina I neurons.

### 2.5. Data analysis

Recordings were taken for quantitative analysis only if the series resistance of the electrode changed during the experiment by less than 20%. Amplitudes of the monosynaptic excitatory postsynaptic current (EPSC) components and EPSC integrals were analyzed with Clampfit software (Molecular Devices, CA, USA). The responses in control and after conditioning were compared for individual cells using Mann–Whitney non-parametric test. For each cell that showed significant differences, the median conditioned value was normalized to the median control one. Then, the data were pooled and presented as mean ± standard error of the mean (SEM). These data were compared using unpaired Student’s *t*-test. Categorical data were compared using Fisher’s exact test. Statistical significance was considered at *p* < 0.05.

## 3. Results

### 3.1. Studying AD-PI

**FIGURE 1:**
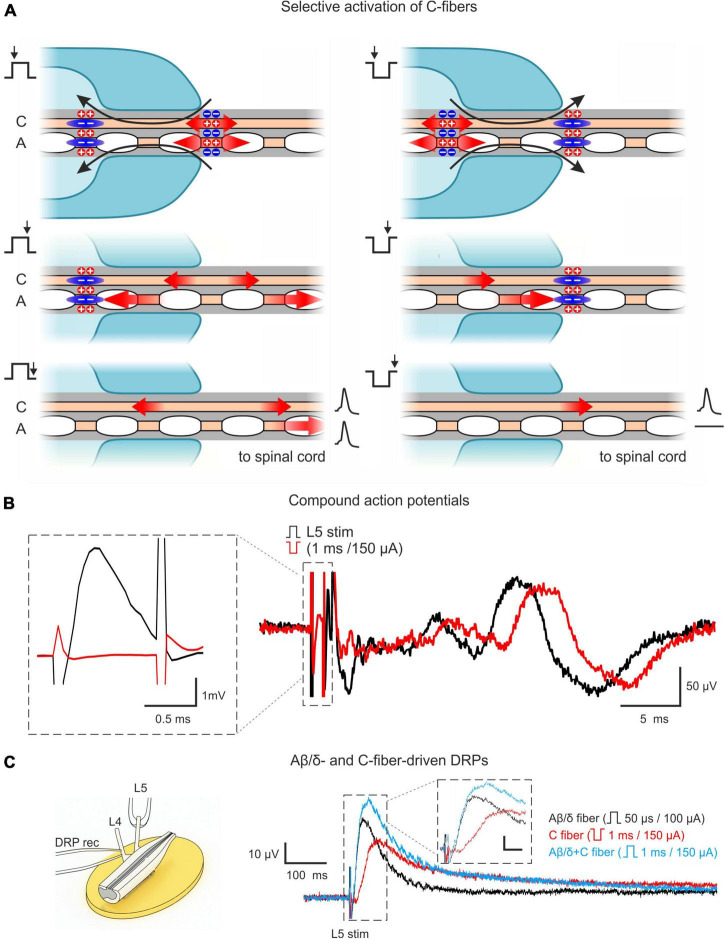
Approach for studying AD-PI in the spinal cord. **(A)** Technique for selective C-fiber activation by the inverted 1 ms pulse stimulation. Red arrows represent the spread of excitation. Blue zones represent regions of hyperpolarization-induced conduction block (anodal block). Given pipette lip geometry and difference in conduction velocities, Aβ/δ-fiber spikes produced by the negative stimuli at the internal end of the pipette lips are blocked at the external end, while C-fiber spikes can propagate to the spinal cord. A detailed description is given in the text. **(B)** C-fiber components of compound action potentials (CAPs) elicited by stimuli of normal (+150 μA × 1 ms, black) and inverted polarity (–150 μA × 1 ms, red). Given the difference in spike initiation sites, C-fiber latency is increased by 2–3 ms for inverted stimuli. The inset shows that a 1 ms stimulus of inverted polarity suppresses the A-fiber-mediated CAP component. **(C)** Dorsal root potential (DRP) recordings from hemisected spinal cord showing Aβ/δ- and C-fiber-driven DRPs. Left: Experimental design. Right: L4 DRPs (averages of 10 traces; recordings from the same preparation) induced by L5 root stimulation activating only Aβ/δ-fibers (+100 μA × 50 μs, black), only C-fibers (–150 μA × 1 ms, red), and Aβ/δ + C-fibers (+150 μA × 1 ms, blue). Inset scale bar: (10 μV, 20 ms). Note that C-fiber-induced DRP peaks at approximately 100 ms.

Our approach relied on two main features. First, we used the *ex vivo* lumbosacral cord preparation which preserved its segmental afferent supply, the circuitries generating AD-PI, and neuronal connections, with an exception of functional descending pathways. Second, selective activation of C-fibers *via* induction of an anodal block of faster-conducting Aβ/δ-fibers. For that, we stimulated the dorsal roots with pulses of inverted (negative) polarity (Figure 1A). A normal (positive) pulse depolarized axons at the external end of the suction electrode lip. Spikes initiated by this local depolarization propagated toward the spinal cord. A pulse of inverted polarity produced depolarization (and spike initiation) at the internal end and hyperpolarization (anodal block) at the external end of the suction electrode lip. The pulse duration (1 ms) was adjusted to induce the anodal block of the Aβ/δ-fiber volley and to terminate before the arrival of the slow C-fiber volley. Thus, only the C-fiber volley could reach the spinal cord. Correct adjustment of the stimulation protocol was confirmed by recording CAPs (Figure 1B).

It should be noted that the major advantage of this approach is that it allows to use one suction electrode and a rectangular current pulse to activate C-fibers and to simultaneously produce an anodal block of the Aβ/δ-fiber volley. This substantially simplified the experimental design in small preparations with relatively short dorsal roots, where induction of the classical anodal block through an additional pair of polarizing electrodes (Mendell and Wall, 1964; Zimmermann, 1968a,b; Wagman and Price, 1969) becomes more difficult.

AD-PI is considered to be induced by PAD, which could be recorded as passively spreading DRP. Therefore, we recorded DRPs to study Aβ/δ- and C-afferent-induced AD-PI. We found that stimulation of both types of afferent evoked DRPs (Figure 1C). Since C-AD DRP peaked at 100–150 ms, the 100 ms interval between paired pulses was used in the experiments with whole-cell recordings to study heterosegmental C-AD-PI.

### 3.2. Homosegmental Aβ/δ-AD-PI

**FIGURE 2:**
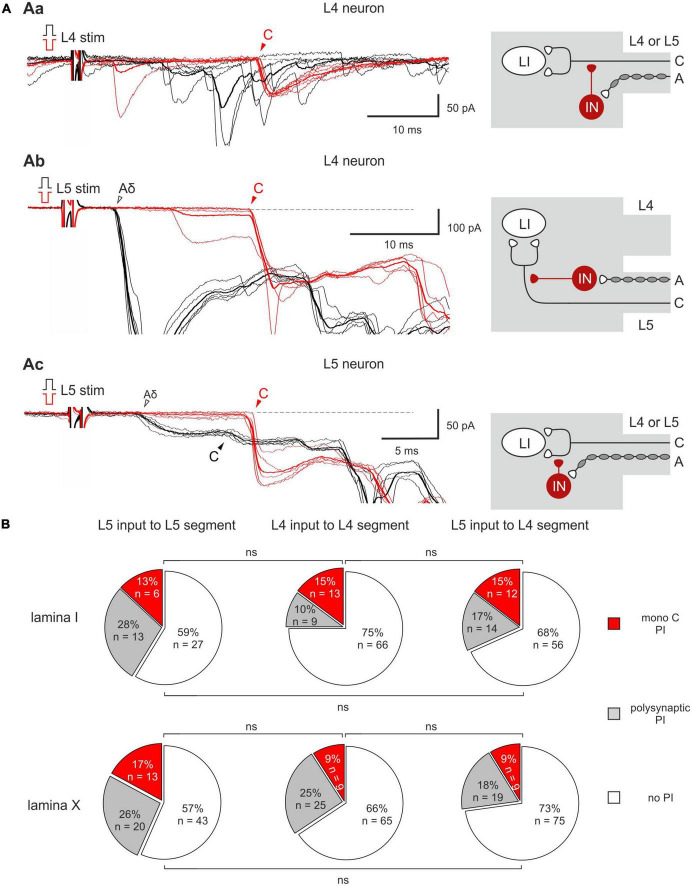
Homosegmental Aβ/δ-AD-PI of C-fiber inputs. **(Aa,b)** Left: complete inhibition of monosynaptic C-fiber inputs. The neuron was located in the segment of the primary afferent entrance **(Aa)** and in the adjacent rostral segment **(Ab)**. Right: putative schematics. **(Ac)** Left: partial suppression of a monosynaptic C-fiber input. Right: putative schematics. Individual (five traces) and averaged (bold) excitatory postsynaptic currents (EPSCs) evoked in spinal neurons by dorsal root stimulation by normal (black) and inverted (red) pulses. Normal pulse (+150 μA × 1 ms) was used to activate all primary afferents, while the pulse of inverted polarity (–150 μA × 1 ms) was applied to induce an anodal block of Aβ/δ-fibers and selectively activate C-fibers. The anodal block of Aβ/δ -fibers resulted in the disappearance of the monosynaptic Aδ-fiber-mediated EPSC (open arrowheads) but either in an appearance of a new monosynaptic C-fiber-mediated EPSC **(Aa,b)** or in an increase in the amplitude of already existing EPSC (**Ac**, note a 2-3 ms increase in the latency of the EPSC evoked by inverted pulse stimulation) which were relieved from AD-PI (filled arrowheads). IN, inhibitory interneuron; LI, lamina I neuron. Putative schemes are valid for both lamina I and X neurons. **(B)** Proportion of lamina I (top row) and lamina X (bottom row) neurons showing C-fiber input disinhibition after anodal block of Aβ/δ-fibers. ns, no significant difference (Fisher’s exact test).

Homosegmental AD-PI is driven by the primary afferents running in the same segmental dorsal root. For Aβ/δ-fibers, this type of AD-PI was assessed using a 1 ms current pulse of normal and inverted polarity. Anodal block of Aβ/δ-fibers by the inverted stimuli disinhibited mono- and/or polysynaptic EPSCs mediated by the homosegmental C-fibers (Figure 2). Disinhibition of the monosynaptic responses occurred in two different ways. In 13–15% of lamina I and 9–17% of lamina X cells, an appearance of a new monosynaptic component (absent in control) reflected a complete Aβ/δ-AD-PI of supplying C-afferents (Figures 2Aa, b). Such form of Aβ/δ AD-PI was seen for the neurons located in the segment of the primary afferent entrance (the L4 afferent input to the L4 neurons, the L5 afferent input to the L5 neurons) as well as in the adjacent rostral segment (the L5 afferent input to the L4 neurons). In 6 lamina I and 3 lamina X cells, we also observed an increase in the amplitude of already existing EPSCs (Figure 2Ac), suggesting the removal of a partial AD-PI affecting one of the terminal C-fiber branches. Disinhibition patterns were similar for the neighboring spinal segments (Figure 2B): the incidence of the L4 and L5 homosegmental Aβ/δ-AD-PI differed significantly neither for lamina I nor for lamina X (*p* > 0.05, Fisher’s exact test). Moreover, the L5 C-fiber input to the L4 neurons was controlled by the L5 Aβ/δ-fibers to a similar extent (*p* > 0.05, Fisher’s exact test). Given that both lamina I and X neurons showed Aβ/δ-AD-PI, one may assume that similar mechanisms control primary afferent input to these two major spinal nociceptive-processing areas.

### 3.3. Heterosegmental C-AD-PI

**FIGURE 3:**
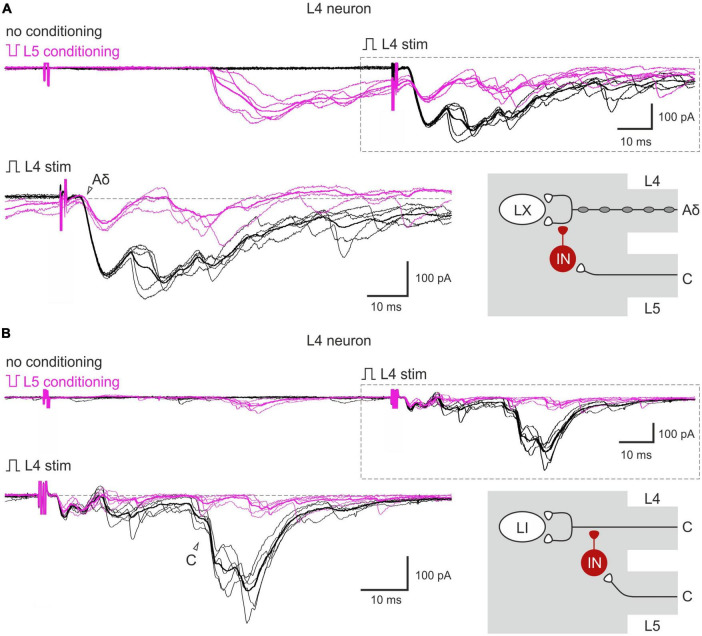
Heterosegmental AD-PI: the L5 C-fibers control the L4 afferent input. The L5 C-fibers induced inhibition of the L4 primary afferent inputs to the L4 spinal neurons. Excitatory postsynaptic currents (EPSCs) were evoked by the L4 root stimulation (normal pulse) in control (black) and after conditioning (100 ms interval) L5 root stimulation (inverted pulse) activating only C-fibers (magenta). The heterosegmental C-fiber conditioning induced a partial **(A)** or full **(B)** suppression of the monosynaptic Aδ- and C-fiber-mediated components of EPSCs (open arrowheads). Individual and averaged (bold) traces are shown. Traces in the insets are shown below at higher magnification and with membrane currents before EPSC initiation set to the same level. Putative schematics of AD-PI given on the right are valid for both lamina I and X neurons. IN, inhibitory interneuron; LI, lamina I neuron; LX, lamina X neuron.

Given that both normal and inverted stimuli activated C-fibers, it was not possible to study interactions between the homosegmental C-afferents. Therefore, we investigated the inhibition of the afferents running in one root by the afferents from the neighboring segmental dorsal root. Paired-pulse protocol was applied to the L4 and L5 dorsal roots while recording from the L4 spinal neurons. We found that selective L5 C-fiber conditioning by inverted pulse significantly diminished the L4 afferent-mediated EPSCs (Figures 3A, B). The amplitudes of the monosynaptic Aδ-components were decreased by 35–40%, while most monosynaptic C-fiber inputs were inhibited completely. The monosynaptic Aβ-fiber input to lamina I neurons was weakly affected; a 10% amplitude reduction was observed in 1 out of 6 cells tested. Thus, heterosegmental C-AD-PI differently affects various classes of primary afferents.

**FIGURE 4:**
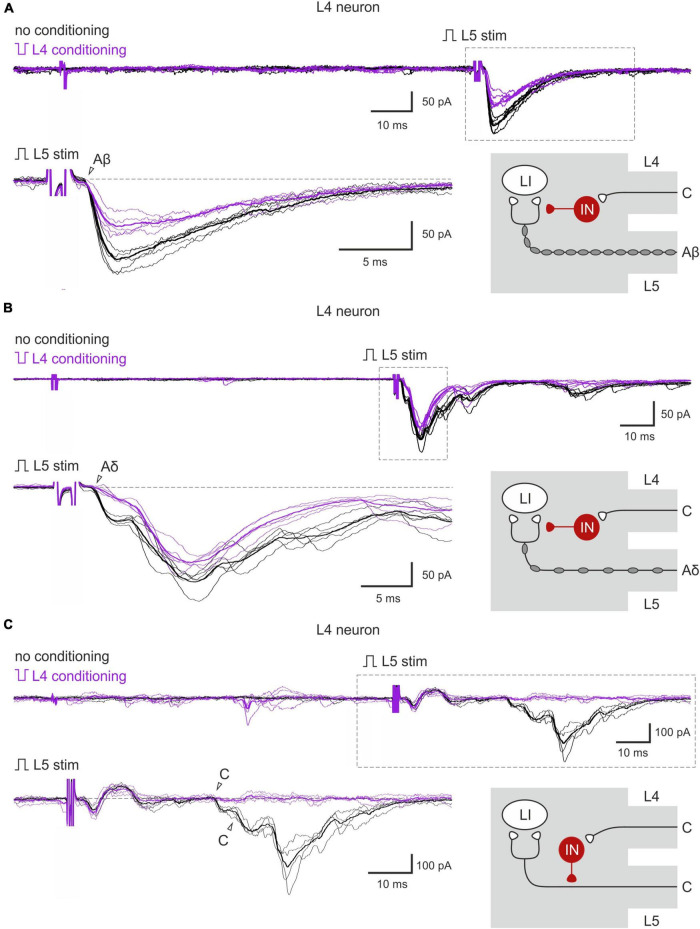
Heterosegmental AD-PI: the L4 C-fibers control the L5 afferent input. **(A–C)** Excitatory postsynaptic currents (EPSCs) evoked by L5 root stimulation (normal pulse) in control (black) and after conditioning (inverted pulse) L4 root stimulation (purple). Individual and averaged (bold) EPSCs are shown. Decreased monosynaptic Aβ- **(A)**, Aδ- **(B)**, and C-components **(C)** of the EPSCs are indicated by open arrowheads. Traces in the insets are shown below at higher magnification. Schematics showing PI induction are valid for both lamina I and X neurons. IN, inhibitory interneuron; LI, lamina I neuron.

**FIGURE 5:**
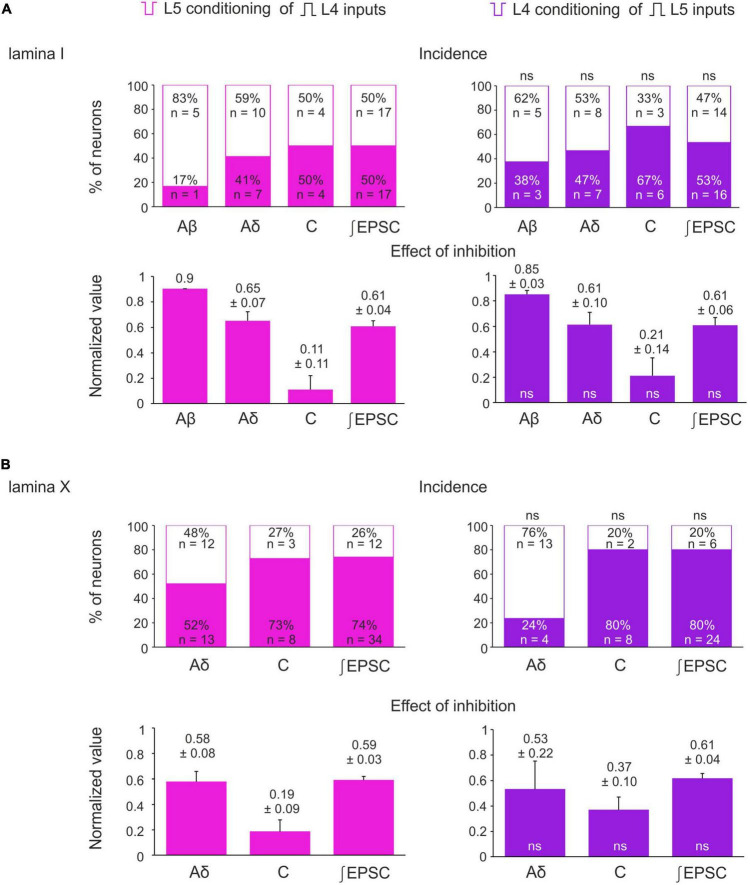
Incidence and strength of the heterosegmental C-AD-PI in spinal laminae I and X. **(A,B)** Top rows: percentage of lamina I neurons **(A)** and lamina X neurons **(B)** showing significant changes in the amplitude of their monosynaptic input and the excitatory postsynaptic current (EPSC) area after heterosegmental C-fiber conditioning. Bottom rows: decrease in the monosynaptic input amplitude and the EPSC area caused by the heterosegmental C-fiber conditioning. Data are represented as mean ± standard error of the mean (SEM). ns, no significant difference when compared to respective parameter from the left column (Fisher’s exact test and Student’s *t*-test for the incidence and the effect of inhibition, respectively).

The inputs from the L5 afferents to the L4 spinal neurons were modulated by the L4 C-fibers in a similar way (Figures 4A–C). Reduction of the monosynaptic Aβ-fiber input (Figure 4A) was observed in 3 out of 8 lamina I neurons and ranged from 11 to 21%. The monosynaptic Aδ responses (Figure 4B) and EPSC integrals were diminished by the C-fiber conditioning to 53–61% and 61%, respectively. Most C-fiber-mediated EPSCs were completely inhibited (Figure 4C). General statistics on the C-fiber-driven control of the afferent input to lamina I and X neurons is presented in Figures 5A, B. Note that for lamina I neurons neither the incidence nor the effect size differed significantly between L4 and L5 heterosegmental C-AD-PI (*p* > 0.05, Fisher’s exact test and Student’s *t*-test, respectively). Similarly, these parameters did not show significant differences for lamina X cells.

**FIGURE 6:**
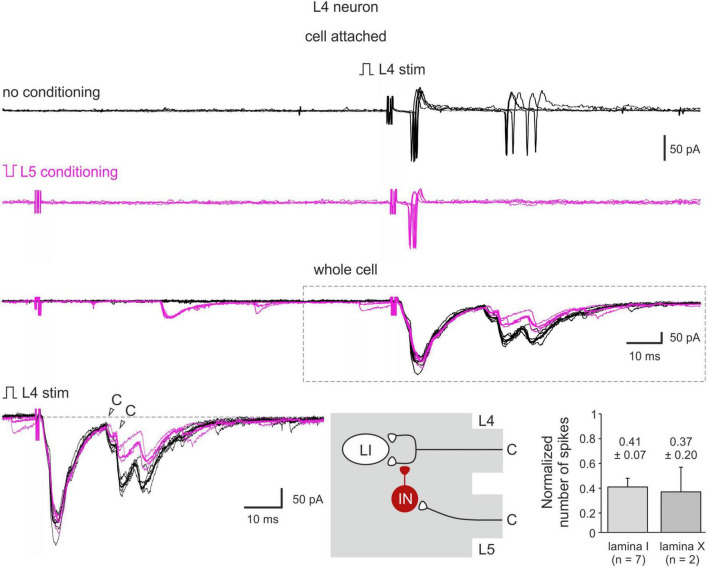
C-AD-PI controls spike discharges in spinal neurons. Cell-attached recording of action potential (AP) firing and the whole-cell recordings of the excitatory postsynaptic currents (EPSCs) evoked by the L4 dorsal root stimulation in a lamina I neuron in control (black) and after the L5 C-fiber conditioning (magenta). Individual (five traces) and averaged (bold) traces of the EPSCs are shown. Bottom middle: schematics showing PI induction. IN, inhibitory interneuron; LI, lamina I neuron. Traces in the inset are shown below at higher magnification. Bottom right: decrease in the number of spikes in lamina I and lamina X neurons.

Heterosegmental C-AD-PI showed its physiological significance in controlling spike discharges in spinal neurons (Figure 6). We tested the effect of the C-fiber conditioning in 15 lamina I and 7 lamina X neurons. In 7 and 2 neurons, respectively, a significant decrease in the overall input and in the number of evoked spikes was observed. On average, the number of spikes was reduced to 41 ± 7% (lamina I) and to 37 ± 20% (lamina X) of control values (Figure 6). Therefore, C-AD-PI is one of the basic mechanisms controlling the excitability of the spinal network.

## 4. Discussion

In the present work, we used *ex vivo* spinal cord preparation with preserved dorsal roots and the technique of selective C-fiber activation to examine the effect of AD-PI on lamina I and X neurons in the mouse and rat. Our major findings are: (1) C-fiber input to the neurons is controlled by both Aβ/δ-AD-PI and C-AD-PI; (2) Aβ/δ-fiber input is subject to C-AD-PI; (3) the AD-PI is segmentally reciprocal, i.e., afferents from two adjacent dorsal roots reciprocally control input to the spinal neurons; (4) C-AD-PI decreases evoked spike discharges, thus demonstrating its physiological significance; and (5) AD-PI pattern is similar for lamina I and X neurons in the mouse and rat. Thus, AD-PI is likely to represent a general mechanism shaping primary afferent input to the neurons in the nociceptive-processing regions of the spinal cord.

The methodological approach we used for studying AD-PI has two principal advantages. First, our *ex vivo* spinal cord preparation preserved its segmental primary afferent supply and neuronal connectivity, with an exception of functional descending pathways. Unlike the spinal cord slices, the intact preparation allowed us to preserve the rostrocaudal and mediolateral connections between the neurons and to work with two adjacent dorsal roots, that was critically important for studying the heterosegmental AD-PI. Second, the dorsal root stimulation by the inverted pulse protocol allowed to selectively activate C-fibers. Thus, we investigated homosegmental Aβ/δ-AD-PI of C-fibers by comparing responses of neurons to the root stimulation by normal and inverted pulses, and the heterosegmental C-AD-PI by using selective C-fiber conditioning by inverted pulses. The main advantages of our approach are its simplicity, and reliability, and that it does not require any genetic modification of the animal model. At the same time, complementing this approach with optogenetic tools allowing specific activation of neurons or primary afferent/descending fibers may bring further benefits to the elucidation of basic mechanisms of PI.

The supraspinal control was shown to play an important role in shaping the functional connectivity between dorsal horn neurons (Contreras-Hernández et al., 2018; Martín et al., 2019). A transection of descending pathways in our preparation, allowed us to study spinal segmental mechanisms in the absence of supraspinal control. We have recently shown that descending PI might have a similar extent as segmental PI (Krotov et al., 2022), suggesting an involvement of the same pool of spinal interneurons. Given functional supraspinal input could seriously interfere with the AD-PI, its absence under our experimental conditions allowed unbiased estimates of the percentage of the AD-PI-affected neurons.

Since the classical gate control theory of pain (Melzack and Wall, 1965) suggested that primary afferents activated by innocuous stimuli affect the transmission of nociceptive information, Aβ/δ-AD-PI of nociceptive C-fibers was well expected. Homosegmental Aβ/δ-AD-PI of the C-fiber input was seen for the neurons in the segment of the dorsal root entrance as well as in the adjacent spinal segment.

The low-threshold Aβ/δ-afferent-mediated postsynaptic inhibition of lamina I neurons plays an important role in controlling their firing threshold and excitability (Luz et al., 2014). However, there are several arguments that the contribution of this and other postsynaptic mechanisms to homosegmental Aβ/δ-AD-PI of the C-fibers observed in our study was unlikely. First, the recordings were done in the voltage-clamp mode, in which inward and outward currents are strictly additive. To exclude the possibility that the monosynaptic C-fiber-mediated EPSC could be reduced by the temporarily coinciding Aβ/δ-fiber-mediated inhibitory postsynaptic current (IPSC) of the same kinetics, we confirmed that the latter was not evoked by selectively activating Aβ/δ-fibers (50 μs stimuli). Thus, the postsynaptic contribution of the GABAergic interneurons that have synaptic contacts with both the central terminals of primary afferents (PI) and spinal neurons (postsynaptic inhibition) (Bardoni et al., 2013; Boyle et al., 2019) could be excluded. Second, we have chosen a 0.1 Hz stimulation frequency to avoid induction of the short-term plasticity or release of kynurenic acid [accumulated in the glial cells (Tuboly et al., 2015)] that could affect the postsynaptic AMPA receptors. Thus, the homosegmental inhibition of C-fibers by Aβ/δ-fibers described here is likely to have a presynaptic nature.

Homotypic C-AD-PI of the C-fiber-induced responses received less attention, despite the fact that C-fibers contribute to the PAD generation (Franz and Iggo, 1968; Zimmermann, 1968a; Zimmerman et al., 2019) and evoke DRPs as strong as those evoked by Aβ/δ-fibers (Zimmerman et al., 2019; Fernandes et al., 2020). The physiological relevance of the C-fiber-driven modulation of other C-fibers is yet to be elucidated, but it might indicate an interplay between C-afferents conveying information about different modalities of nociceptive information. The same might be the case for C-AD-PI of Aβ/δ-fiber input. Aδ-afferents (Mackenzie et al., 1975; Simone and Kajander, 1997; Todd, 2010) and, to a lesser extent, Aβ-afferents (Djouhri and Lawson, 2004; Nagi et al., 2019) are also involved in the transmission of noxious stimuli. However, these forms of AD-PI may reflect the presence of complex neuronal networks controlling the processing of noxious and innocuous primary afferent inputs.

AD-PI in lamina I has a plausible mechanistic explanation. Axo-axonic synapses, generally considered as a structural basis for PI (Conradi et al., 1983), target C-fiber terminals forming type I glomeruli in the middle and ventral parts of lamina II (Ribeiro-Da-Silva and Coimbra, 1982; Ribeiro-da-silva et al., 1989; Todd, 1996)—a zone to which ventral dendrites of lamina I neurons protrude (Fernandes et al., 2016, 2020). Some local circuit interneurons have dendrites extending up to lamina III (Fernandes et al., 2016) where type II glomeruli (formed by myelinated A-afferents) are abundant (Ribeiro-Da-Silva and Coimbra, 1982). Glomerular PI can suppress transmission in individual synapses and produce a partial suppression of C- and A-afferent input.

On the other hand, glomeruli are not reported in lamina X. Moreover, lamina I is practically devoid of glomeruli or simple axo-axonic synapses (Ribeiro-Da-Silva and Coimbra, 1982; Ribeiro-da-silva et al., 1989; Alvarez et al., 1993), despite being enriched with Aδ- and C-fiber terminals (Todd, 2010). Although rare axo-axonic synapses might be sufficient for AD-PI induction (Segev, 1990), some other glomeruli-independent mechanisms of AD-PI generation should also be considered.

Non-synaptic AD-PI mechanisms may involve GABA_A_ receptors, expressed in the central axons and terminals of thin afferents (Knabl et al., 2008; Witschi et al., 2011; Paul et al., 2012; Lorenzo et al., 2014). These receptors might be activated *via* volume transmission by GABA released from interneurons or astrocytes (Christensen et al., 2018; Comitato and Bardoni, 2021). Alternatively, shunting inhibition might be mediated by AMPA and NMDA receptors (Russo et al., 2000; Comitato and Bardoni, 2021), which are also expressed in the central terminals of primary afferents (Liu et al., 1994; Bardoni et al., 2004). All these mechanisms may contribute to the suppression of signals in the non-glomerular terminal boutons or the parent branches of primary afferents. This can explain the complete inhibition of EPSC components observed in our experiments. The non-synaptic mechanism may play a major role in the induction of AD-PI in lamina X neurons and in Aδ- and peptidergic C-fibers that terminate in lamina I (Todd, 2010).

Despite the obvious physiological importance of AD-PI, we still have limited knowledge about its segmental organization. AD-PI induced by stimulating L4 and L5 roots exhibits segmental reciprocity that could be expected from earlier studies of DRPs (Wall and Lidierth, 1997; Lidierth, 2006). In both laminae I and X, inputs from these two adjacent roots are under reciprocal control. This feature might be relevant for the spatial discrimination of stimuli or for avoiding an excessive excitation when the input from one peripheral point arrives *via* adjacent dorsal roots (Pinto et al., 2008b,^2010^). Furthermore, C-AD-PI controls spike discharge evoked by the afferent stimulation, indicating that this form of inhibition has a direct impact on the activity of spinal neuronal circuitries. It should be noted that in our experiments the whole dorsal roots were stimulated, and therefore, the reciprocity and functional impact observed reflect afferent interactions at the segmental level. This might not necessarily mean that the reciprocal inhibition occurs and/or has a functional impact at the level of individual afferents. More specific ways of physiological stimulation of different classes of primary afferents would be needed to clarify this issue.

Our study describes how AD-PI affects peripheral inputs to spinal neurons in laminae I and X. For lamina I, the pattern of the AD-PI induction is similar for the mouse (our study) and rat (Fernandes et al., 2020), suggesting a fundamental role of AD-PI in nociceptive processing in mammalians. There is virtually no difference in how the AD-PI affects the neuron populations in laminae I and X; they both exhibit the L4/L5 segmental reciprocity and AD-PI-mediated control of neuronal spike discharges. This may be explained by the fact that both these layers are involved in nociceptive-processing and their neurons show similar intrinsic properties and primary afferent supply (Light and Perl, 1979; Honda, 1985; Honda and Perl, 1985; Fernandes et al., 2016; Krotov et al., 2019). Presynaptic control of the afferent input occurs in a substantial percentage of cells. This observation was done in the experiments in which the whole dorsal root was stimulated and may reflect the fact that AD-PI broadly affects the spinal sensory network. At the same time, AD-PI is not ubiquitous, suggesting that it can control processing of sensory information in specific cell circuitries.

## Data availability statement

The raw data supporting the conclusions of this article will be made available by the authors, without undue reservation.

## Ethics statement

The animal study was reviewed and approved by the Animal Ethics Committee of the Bogomoletz Institute of Physiology (Kyiv, Ukraine).

## Author contributions

VK: concept of the study, research design, electrophysiological recordings, data analysis and interpretation, and manuscript preparation. KA, SR, OH, and YA: electrophysiological recordings and data analysis. BVS: manuscript preparation and critical revision. PB and NV: conceiving the study and manuscript revision. All authors contributed to the article and approved the submitted version.
